# Effect of one-time high load exercise on skeletal muscle injury in rats of different genders: oxidative stress and mitochondrial responses

**DOI:** 10.1590/acb370805

**Published:** 2022-12-12

**Authors:** Yuan Wang, Mengmeng Chen, Yan Gao, Kang He, Zhaoyun Yang, Yuewei Li, Shuang Zhang, Lijing Zhao

**Affiliations:** 1 Master. Jilin University – School of Nursing – Department of Rehabilitation – Professional Master’s Program – Changchun, China.; 2 Bachelor. Jilin University – School of Nursing – Department of Rehabilitation – Professional Bachelor’s Program – Changchun, China.; 3 PhD, Associate Professor. Jilin University – School of Nursing – Department of Rehabilitation – Changchun, China.

**Keywords:** Exercise, Muscle, Skeletal, Injury, Gender

## Abstract

**Purpose:**

To evaluate the impact of one-time high load exercise on skeletal muscle injury and analysis its mechanism in different genders.

**Methods:**

Twenty-four male and 24 female rats were divided randomly into four groups respectively: control, 0 h, 6 h, and 24 h after exercise. The activities of creatine kinase (CK), lactate dehydrogenase (LDH), and myohemoglobin (MYO) in serum, the expression level of oxidative stress markers, mitochondrial respiratory chain complex enzyme, and the apoptosis related protein in quadriceps were detected.

**Results:**

The results showed that the activities of CK, LDH and MYO in serum increased immediately after exercise and restored faster in female rats. More obvious structural disorder and apoptosis in male rats were showed. Malondialdehyde (MDA) and superoxide dismutase (SOD) were increased while catalase (CAT) and glutathione (GSH) were decreased in male rats. SOD, CAT and GSH were increased in female rats. Mitochondrial complex enzyme activity was decreased in males and increased in females.

**Conclusions:**

The skeletal muscle injury in both genders of rat could be induced by one-time high load exercise due to the mitochondrial respiratory enzyme dysfunction and oxidative stress, which was relatively mild and recovered quicker in female rats.

## Introduction

As a method of clinical rehabilitation therapy, exercise has been widely used and appreciated. According to the definition of the American College of Sports Medicine, exercise refers to planned, structured, repeatable physical activity that can improve and maintain one or more physical fitness components, and is an important part of healthy life^1^. However, due to the relative lag in the popularization and education of healthy sports knowledge, accidental injuries during sports emerge in an endless stream. To a certain extent, exercise has brought people into health risks, and exercise -injury is no longer the exclusive term of athletes. Among them, muscle injury induced by high-load exercise is the most common and worthy of attention^2^. Some studies have found that the degree of high-load exercise injury varies by gender^3^^,^ 4, but the specific mechanism of this phenomenon has not been conclusive due the lack of systematic theoretical research.

Studies have shown that exercise promotes oxidative stress in the body^5^, and that high-intensity exercise, both long and short, can lead to increased free radical production in active skeletal muscles, resulting in the formation of oxidized lipids and proteins in the working muscles^6^^–^ 8. This uncontrolled production of reactive oxygen species (ROS) can damage cells. In addition, increasing evidence reveals that although uncontrolled production of reactive nitrogen species and ROS can damage cells, intracellular oxidants play important roles in regulating the modulation of skeletal muscle force production, regulating cell signaling pathways, and controlling gene expression^9^^–^ 11.

ROS can affect the structure and function of mitochondria, the main site of cellular energy metabolism, resulting in increased mitochondrial membrane permeability and disturbance of electron transport system of the respiratory chain^12^^,^ 13. On the other hand, ROS is mainly produced generated during the erroneous electron transfer of mitochondrial respiratory chain complex enzyme^14^. Therefore, disturbance of ROS and mitochondria may be the underlying mechanism high-load exercise injury.

## Methods

Forty-eight 8-week-old Wistar rats, half male and half female, were purchased from Beijing Unilever Laboratory Animal Co., Ltd. The feeding and operation of experimental animals followed the Guidelines of the Laboratory Protocol of Animal Care and Use Committee of Jilin University.

After one week of adaptive feeding, the rats of each gender were randomly divided into 4 groups of 6 rats: control group (Ctr), 0 h after exercise (0 h), 6 h after exercise (6 h), and 24 h after exercise (24 h). After 10 min of acclimatization exercise every day for 3 consecutive days, the rats in the exercise groups exercised on the treadmill at a speed of 20 m/min for 90 min, while the rats in the control groups exercised freely on the treadmill for 90 min. The load strength was based on the study results of Bedford *et al*.^15^. If the rats were exhausted during the exercise, let them rest for 2 min before continuing to ensure the total exercise time. The creatine kinase (CK) levels were also measured to assist in determining muscle damage after one-time high-load exercise.

### Blood and tissue preparation

The rats (0 h, 6 h and 24 h after exercise, respectively, including the control group) were euthanized with intraperitoneal injection of pentobarbital sodium (30 mg/kg). Blood was collected from the abdominal aortas and centrifuged at 3,000 g for 10 min at 4 °C, and the serum was stored at –80 °C for lactate dehydrogenase (LDH), myohemoglobin (MYO) and CK analysis. The quadriceps were dissected, soaked in formaldehyde or frozen at –80 °C for further analysis. Ten percent muscle homogenate was prepared for malondialdehyde (MDA), glutathione (GSH), catalase (CAT) and superoxide dismutase (SOD) detection.

### Biochemical measurements

Activities of LDH (A020-2-2) and CK (A032) in serum were determined according to kit instructions (Nanjing Jiancheng, Nanjing China). Concentration of MYO (E-EL-R0053c) in serum was measured with ELISA kit (Elabscience, Wuhan, China). Level of MDA (A003-1-2) and GSH (A006-2-1), and activities of CAT (A007-1-1) and SOD (A001-1-2) were determined by commercial kits (Nanjing Jiancheng, China).

### Hematoxylin and eosin staining for histopathological evaluation

Rat quadriceps were fixed with 4% paraformaldehyde at room temperature (RT) for 24 h, embedded in paraffin, and sliced into sections with a thickness of 4 μm. Histopathological changes were examined after hematoxylin and eosin (H&E) staining^16^.

### Terminal deoxynucleotidyl transferase dUTP nick end labeling (TUNEL) staining

The deparaffinized sections of quadriceps were stained with TUNEL detection kit (GDP1042, Servicebio, Wuhan, China). Then, the slides were observed under a fluorescence microscope, and images were captured with digital camera (BX51, Olympus, Japan). The apoptotic index (positive cells / total cells × 100%) was calculated from five randomly visions in each section.

### Immunohistochemistry

The quadriceps sections were incubated with 3% methanol hydrogen peroxide to block endogenous peroxidase for 30 min at RT, preincubated in 2% bovine serum albumin (BSA) for 30 min to block nonspecific reactivity, incubated with antinuclear factor erythroid 2-related factor 2 (Nrf-2) overnight at 4 °C, processed with biotinylated secondary antibodies for 1 h at RT, incubated with streptavidin-horseradish peroxidase for 10 min at RT in chronological order. Immunoreactive products were visualized in 0.03% H_2_O_2_ and 0.05% diaminobenzidine. The sections were dehydrated, cleared, mounted and observed under light microscope.

### Mitochondrial complex enzyme I, II, IV activity detection

Quadriceps pieces of 100 mg were mixed with 1.0 mL of extract, homogenized on ice, and centrifuged at 600 g at 4 °C for 10 min. The supernatant was transferred to another centrifuge tube, and centrifuged at 11,000 g at 4 °C for 15 min. The precipitate was dissolved in 400 μL extract, then crushed with ultrasonic at 20% power, coupling 5 s ultrasonic and 10 s interval for 15 times. The activities of complex I (BC0515), II (BC3235) and IV (BC0945) were determined by instructions of the Electron Transport Chain Complex assay Kit (Solarbio, Beijing, China).

### Western blot analyses

Protein from 100 mg quadriceps was lysed in RIPA and PMSF buffer (100:1). The total protein was quantified by the Bradford method (P0006C, Beyotime, Shanghai, China). The equivalent amount of 30 μg protein were separated in 10% SDS-PAGE (Servicebio, Wuhan, China) and transferred to polyvinylidene difluoride membranes (IPVH00010, Millipore, USA). The membranes were blocked with 5% (w/v) nonfat milk in TBST (100 mmol L^-1^ Tris, 1.5 mmol L^-1^ NaCl, pH 8.0 and 0.5% Tween 20), incubated with primary antibodies in 5% BSA in TBST overnight at 4 °C, and incubated with secondary horseradish peroxidase-conjugated anti-Rabbit IgG (SA00001-2, 1:4,000, Proteintech, USA) diluted in TBST. Immunoreactive bands were detected by enhanced chemiluminescence. Images were quantified by Image-J (National Institutes of Health, USA).

Primary antibodies used in this study include: anti-GAPDH (AF7021, 1:2,000, Affinity, UK), anti-tubulin (bs-20694R, 1:1000, Bioss, China), anti-COX4 (11242-1-AP, 1:1,000, Proteintech, USA), anti-Bcl-2 (12789-1-AP, 1:1,000, Proteintech, USA), anti-Bax (50599-2-Ig, 1:1,000, Proteintech, USA), anti-caspase 9 (AF6348, 1:1,000, Affinity, UK), anti-caspase 3 (AF6311, 1:1,000, Affinity, UK), antinuclear factor erythroid 2-related factor 2 (Nrf2, AF0639, 1:1,000, Affinity, UK), anti-Keap1 (AF5266, 1:1,000, Affinity, UK), anti-heme oxygenase-1 (HO-1, AF5393, 1:1,000, Affinity, UK), anti-NADH quinone oxidoreductase 1 (NQO1, DF6437, 1:1,000, Affinity, UK), anti-glutathione peroxidase (GPX4, 1:1000, Proteintech, USA), anti-ATP5f1 (DF12144, 1:1,000, Affinity, UK) anti-cytochromec1 (CYC1, 10242-1-AP, 1:1,000, Proteintech, USA), anti-NADH dehydrogenase ubiquitin flavin 1 (NDUFV1, bs-3959R, 1:1,000, Bioss, China). All antibodies were diluted by TBST and all manipulations strictly followed the manufacturer’s standard protocol.

### Statistics

The data was analyzed by SPSS ver. 26.0 (IBM, USA) and expressed in mean and standard deviation (x_ ± s). One-way analysis of variance (ANOVA) was applied to compare the differences among different groups. All experimental results were verified independently for more than three times. The differences were considered significant at p < 0.05.

## Results

### One-time high-load exercise induced muscle injury

**Figure 1:**
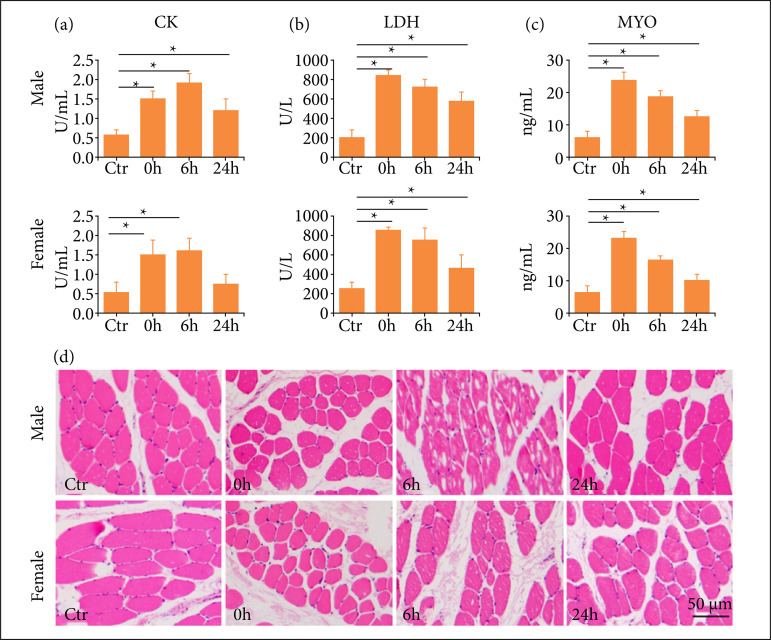
One-time high-load exercise induce muscle injury in rats; **(a)** the activity of serum CK;**(b)** The activity of serum LDH; **(c)** The concentration of serum MYO; **(d)** The histologicalchanges of muscles by H&E staining. *p < 0.05 compared with control group.

To evaluate the injury of skeletal muscles due to exercise, the biochemical parameters related to cell injury including serum CK, LDH and MYO were detected. The results showed that these biomarkers of muscle injury were elevated after one-time high-load exercise compared to control groups. The activity of CK increased significantly from immediately to 6 h after exercise, decreased at 24 h after exercise, and the decrease in female rats was more pronounced compared with male rats (Fig. 1a). The activity of LDH and the concentration of MYO were increased immediately after exercise and decreased gradually over time, and were lower significantly in female rats compared with male rats in 24 h groups (Fig. 1 b,c). To further assess the influence of one-time high-load exercise on muscles, pathological changes were observed after H&E staining. As showed in Fig. 1d, the muscle fibers in the control groups were neatly arranged, with clear structure and uniform distribution of nuclei. The muscle fiber gap widened at 0 h after exercise, the disorder of muscle fibers and the vacuolar-like changes in muscle cells were observed at 0 h after exercise, and the injury degree of male rats was more serious than that of female rats. The histological changes were significantly recovered at 24 h after exercise.

### One-time high-load exercise induced apoptosis

**Figure 2:**
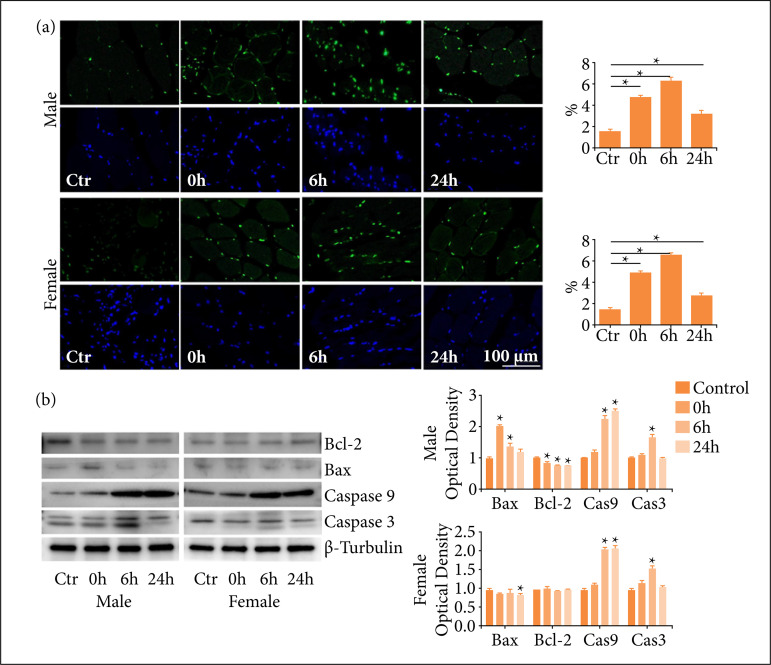
One-time high-load exercise induce apoptosis of muscle; **(a)** Apoptosis detectedby TUNEL staining; **(b)** Expression of apoptosis-related protein detected byWestern blot. * p < 0.05 compared with the control group.

In order to investigate the possible mechanism of the muscle injury induced by exercise, TUNEL staining was used to test the apoptosis of muscle cells. The results showed that TUNEL-positive muscle cells increased significantly up to 6 h after exercise compared with control groups, and more positive cells were observed in male rats than female rats (Fig. 2a). To further evaluate the apoptosis of myocytes, the expression of apoptosis-related protein was detected by Western blot. After exercise, the expressions of caspase 9 increased immediately at 0 h and maintain the high level until 24 h in males as well as female rats. The expression of caspase 3 increased significantly at 6 h and then decreased at 24 h. The expression of Bax increased significantly after exercise in male rats, but there was no significant change in female rats before and after exercise. In male rats, Bcl-2 expression decreased in a time-dependent manner after exercise, but there was no significant change in female rats (Fig. 2b).

### One-time high-load exercise induced oxygen stress

**Figure 3:**
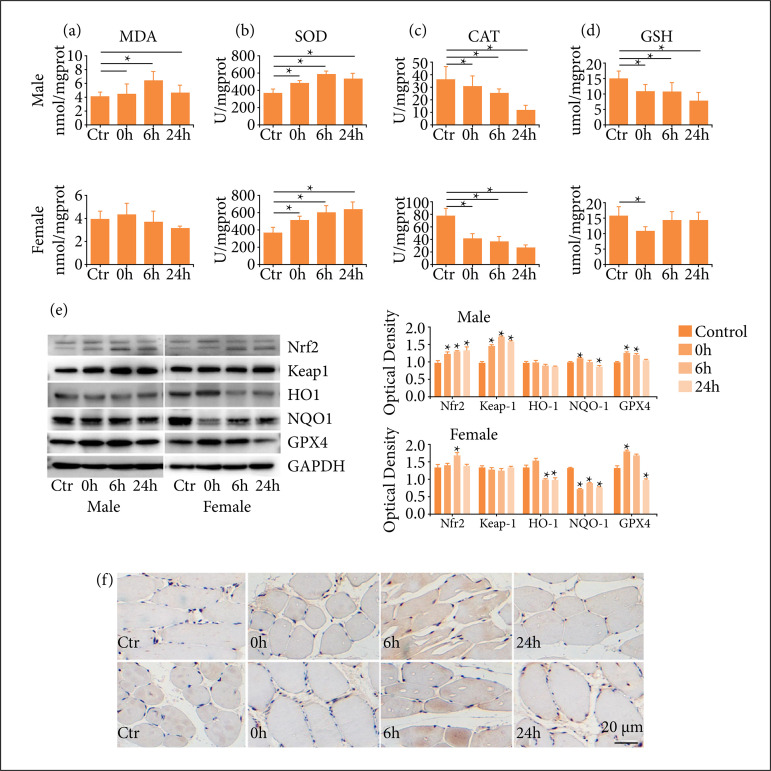
One-time high-load exercise induce oxygen stress; **(a)** The concentration of MDAin quadriceps; **(b)** The activity of SOD in quadriceps; **(c)** The activity of CAT in quadriceps;**(d)** The activity of GSH in quadriceps; **(e)** The expression antioxidant protein inquadriceps; **(f)** The location of Nrf2. * p < 0.05 comparedwith the control group.

To detect whether the apoptosis was induced by oxidative stress, the concentration of MDA and the activities of SOD, CAT and GSH in quadriceps were tested. The results showed that MDA level increased at 6 h after exercise in male rats, and then decreased, but no significant differences were observed in the four groups of female rats (Fig. 3a). For female rats, the activity of SOD increased gradually, and still maintained high level at 24 h, while that of male rats reached the highest level at 6 h after exercise, and decreased at 24 h after exercise (Fig. 3b). The activity of CAT increased at 6 h and then decreased at 24 h after exercise in female rats, while decreased in time-dependent manner in male rats (Fig. 3c). The activity of GSH after exercise decreased both in male and female rats, and the decrease was more obvious in male rats (Fig. 3d). To further evaluate oxidative stress response induced by one-time high-load exercise, the expression of oxidative stress related protein was detected. As showed in Fig. 3e, the expression of Nrf2 and Keap1 increased after exercise in male rats, but just Nrf2 increased at 6 h after exercise in female rats. The expression of HO-1 decreased at 6 and 24 h in female rats, but no obvious change was detected in male rats after exercise. The expression of NQO1 increased in male rats, but decreased in female rats. The expression of GPX4 increased after exercise immediately, and decreased at 24 h after exercise in both genders (Fig. 3e). Furthermore, the results of immunohistochemical staining showed that Nrf2 was distributed mainly in the cytoplasm of muscle cells in the control group, and transferred to the nucleus after exercise (Fig. 3f).

### One-time high-load exercise induce dysfunction of mitochondria

**Figure 4:**
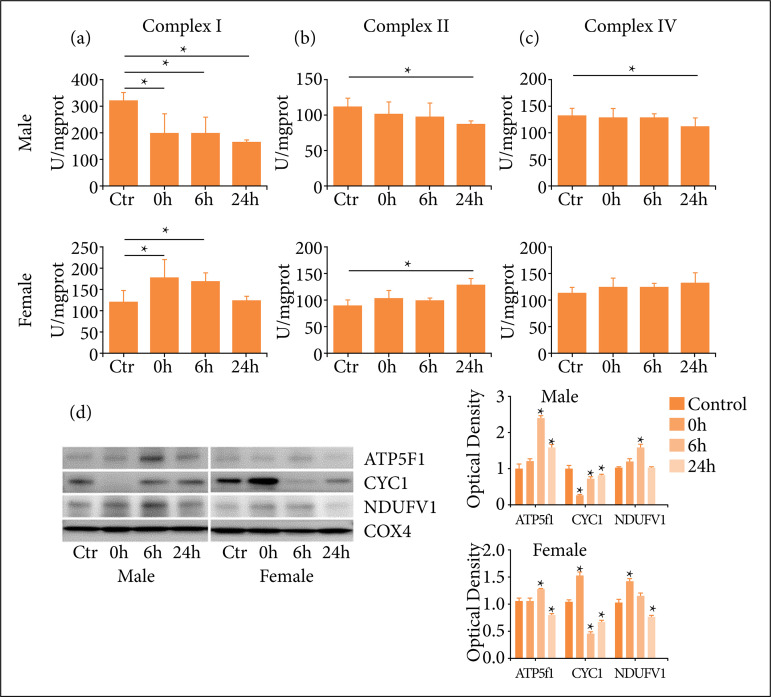
One-time high-load exercise induce dysfunction of mitochondria; **(a–c)** Activitiesof mitochondrial complex I, II and IV; **(d)** Differential expression of mitochondrialproteins. * p < 0.05 compared with the control group.

ROS is mainly induced in mitochondrion respiratory chain during electric transportation, in which mitochondrial complexes play an important role. In this study, mitochondrial complex enzyme was detected to verify the influence of one-time high-load exercise on the function of mitochondria. The results showed that the activities of mitochondrial complex enzyme I, II and IV in male rats decreased after exercise. The activities of enzyme I and II increased in female rats, while the activity of enzyme IV increased slightly without statistical significance (Fig. 4 a–c). Furthermore, the expression of mitochondrial complex protein was detected in this study. As shown in Fig. 4d, in male rats, the expression of NDUFV1 and ATP5f1 increased at 6 h, and decreased at 24 h after exercise, while CYC1 decreased at 0 h after exercise, and then increased gradually. In female rats, the expressions of NDUFV1 and CYC1 increased at 0 h, and then decreased, and the expression of ATP5f1 increased at 6 h after exercise.

## Discussion

Intense exercise can affect the permeability of cell membrane, allowing some macromolecules in the cytoplasm to leak out through the cell membrane and enter the blood^17^^,^ 18. Therefore, the levels of these macromolecules in serum can be used to judge cellular injury. This study found that after one-time high-load exercise, the levels of CK, LDH and MYO in the rat serum were increased, indicating that muscle damage induced by high-load exercise. It seemed that the leakage of female rats was less than that in male rats, which might be attributed to the membrane stabilization affected from estrogen. The H&E staining displayed less morphological change of skeletal muscle cells in female rats. Furthermore, the results of TUNEL staining and apoptotic related protein expression indicated that the muscle damage was induced at least partly by cell apoptosis.

Previous study has confirmed that high-intensity exercises could induce more free radicals in active skeletal muscle, resulting in the formation of oxidized lipids and proteins in working muscle^19^. Among the aldehydes derived by lipid peroxidation, MDA is used as a valid marker to monitor the peroxidation of polyunsaturated fatty acids. In this study, elevated MDA levels indicated that more oxygen was took up during exercise, leading to increased production of free radicals and putting cells in a state of oxidative stress.

Researchers have proposed that the activation of Nrf2, a key factor in cellular oxidative stress response, may be a means of regulating intracellular pathways related to ROS detoxification^20^. Therefore, the expression and location of Nrf2 in skeletal muscle was detected in this study, and the upregulation and nucleus translocation of Nrf2 were observed especially in male rats, indicating that metabolic reprogramming and protection occurred in parallel during exercise, resulting in enhanced antioxidant defense and protection against lipid peroxidation^21^^–^ 23.

Under stress conditions such as ROS, Nrf2 is phosphorylated, uncoupled from Keap1, translocated into the nucleus, interacted with antioxidant response elements to regulate the expression of antioxidant enzyme such as SOD, CAT and GSH, and phase II detoxification enzyme such as HO-1 and NQO-1. Although uncontrolled production of ROS can damage cells, intracellular oxidants play important regulatory roles in the production of skeletal muscle force, cell signaling pathways, and control of gene expression^6^^,^ 24 ^,^ 25. In this study, the compensatory increase of SOD enzyme activity suggested that high-load exercise led to an increase in free radicals in the body, but the body increased the expression of antioxidant enzyme and SOD activity, thereby inhibiting the effect of free radicals on cells damage^26^. CAT could catalyze the breakdown of hydrogen peroxide into oxygen and water, thereby reducing lipid peroxidation. GSH is a sulfhydryl-containing tripeptide acting antioxidant and comprehensive detoxification effects. In this work, the activities of CAT and GSH after exercise were significantly decreased in male rats, suggesting that excessively produced free radicals contribute to increase consumption of them. In female rats, the increasing of CAT suggested that females had stronger antioxidant capacity, and the enhanced activity of CAT might be related to estrogen^27^^,^ 28.

GPX4 can reduce the content of H_2_O_2_ in cells by oxidizing GSH, thereby effectively scavenges free radicals in the organism^29^^–^ 31. Therefore, GPX4 expression is often inversely correlated with GSH activity. In this study, the expression of GPX4 in muscle was significantly increased after exercise, while GSH activity was decreased, indicating that GPX4 was actively involved in lipid peroxidative scavenging in skeletal muscle, especially in males, which illustrated a higher degree of lipid peroxidation in male rats after exercise.

It has been reported that HO1 have anti-inflammatory and antioxidant properties, thereby protecting against oxidative stress injury and inhibiting apoptosis^32^. NQO1, a reductive coenzyme I/II-dependent flavoprotein, catalyzes the loss of two electrons from quinones and their derivatives as an acceptor, resulting in a reduction reaction, thereby reducing the formation of intermediate semiquinones and oxidation products to avoid cell damage^33^^–^ 35. In this study, the expressions of HO1 and NQO1 in male rats increased after exercise, indicating that the free radicals generated by exercise stimulate the activation of Nrf2 and then upregulate the expressions of HO1 and NQO1. However, there were no significant change in the expression of HO1 and NQO1 in female rats, indicating that female rats produced fewer free radicals than male rats after exercise, and thus the regulation of HO-1 and NQO1 expression were weaker, which were consistent with the change in MDA.

The respiratory chain, located in the inner membrane of mitochondria, performs electron transport and generates ATP. The function of respiratory chain is mainly completed by five complex enzymes, whose structural damage or activity change will affect the respiratory function, resulting in a large accumulation of free radical. Studies have showed that adaptive change in mitochondria can be induced by exercise, such as the increasing of enzyme synthesis, activity, and oxidative phosphorylation^36^^,^ 37.

The results of this study showed that the activities of mitochondrial complexes I, II and IV were decreased in male rats after exercise, indicating that energy metabolism in skeletal muscle decreased, leading to mitochondrial damage. The activities of complex enzymes I and II increased after exercise in female rats, indicating that the respiratory chain of female rats had significant adaptive ability, which could enhance the anti-injury function by increasing the activity of complex enzyme and improving mitochondrial function. And the results of mitochondrial complex protein expression showed that NDUFV1 and ATP5F1 were both up-regulated by exercise, and CYC1 was down-regulated in male rats, while up-regulated in female rats. The NADH dehydrogenase ubiquitin flavin 1 encoded by NDUFV1 is a subunit of complex I with NADH, FMN and Fe-S binding sites. CYC1 is a subunit of complex III that accepts iron from Fe-S and transfers electrons to cytochrome C. ATP5F1 is a subunit of ATP synthase that drives protons back to the substrate through the H^+^ gradient of the inner mitochondrial membrane. The results suggested that exercise could affect mitochondrial function, lead to cellular metabolic dysfunction and cause oxidative stress.

## Conclusion

This study preliminarily demonstrated the injury effects and mechanism of one-time high-load exercise on rat skeletal muscle. The results showed that high-load exercise led to attrition of skeletal muscle mitochondria, decreased activity of respiratory chain complex enzyme, increased ROS production, and induced cell damage. Female rats suffered less damage due to better adaptability. Therefore, the injury effect of sports and gender differences should be considered when formulate training plans for athletes or exercise prescriptions for patients, and improving mitochondrial function can be used for preventing exercise injury.
